# Possible interference between seasonal epidemics of influenza and other respiratory viruses in Hong Kong, 2014–2017

**DOI:** 10.1186/s12879-017-2888-5

**Published:** 2017-12-16

**Authors:** Xueying Zheng, Zhengyu Song, Yapeng Li, Juanjuan Zhang, Xi-Ling Wang

**Affiliations:** 1 Department of Biostatistics and Key Laboratory of Public Health Safety, School of Public Health, Fudan University, Shanghai, China; 2 Collaborative Innovation Center of Social Risks Governance in Health, Fudan University, Shanghai, China; 3 Shanghai Key Laboratory of Meteorology and Health, Shanghai, China

**Keywords:** Influenza, Interference, Respiratory viruses, Seasonal epidemics

## Abstract

**Background:**

Unlike influenza viruses, little is known about the prevalence and seasonality of other respiratory viruses because laboratory surveillance for non-influenza respiratory viruses is not well developed or supported in China and other resource-limited countries. We studied the interference between seasonal epidemics of influenza viruses and five other common viruses that cause respiratory illnesses in Hong Kong from 2014 to 2017.

**Methods:**

The weekly laboratory-confirmed positive rates of each virus were analyzed from 2014 to 2017 in Hong Kong to describe the epidemiological trends and interference between influenza viruses, respiratory syncytial virus (RSV), parainfluenza virus (PIV), adenovirus, enterovirus and rhinovirus. A sinusoidal model was established to estimate the peak timing of each virus by phase angle parameters.

**Results:**

Seasonal features of the influenza viruses, PIV, enterovirus and adenovirus were obvious, whereas annual peaks of RSV and rhinovirus were not observed. The incidence of the influenza viruses usually peaked in February and July, and the summer peaks in July were generally caused by the H3 subtype of influenza A alone. When influenza viruses were active, other viruses tended to have a low level of activity. The peaks of the influenza viruses were not synchronized. An epidemic of rhinovirus tended to shift the subsequent epidemics of the other viruses.

**Conclusion:**

The evidence from recent surveillance data in Hong Kong suggests that viral interference during the epidemics of influenza viruses and other common respiratory viruses might affect the timing and duration of subsequent epidemics of a certain or several viruses.

## Background

The substantial morbidity, mortality rates and economic burden of influenza and other respiratory illness have been well discussed globally [1–5]. Influenza affects 5%–15% of the population and causes 250,000 to 500,000 deaths annually worldwide [6]. The cost of one influenza-like (ILI) case, which includes the costs of medical care and days lost due to ILI, represents approximately about 20% of the monthly per capita income in southern China [2]. By contrast, studies on the recent aetiology and circulation of multiple respiratory illnesses circulating concurrently amongst the population in regions of China are limited [7]. Detected by the ILI surveillance system, the common viruses that cause respiratory illness symptoms in Hong Kong and other regions include, but are not limited to, influenza viruses, respiratory syncytial virus (RSV), parainfluenza virus (PIV), adenovirus, enterovirus and rhinovirus [8, 9].

The existence of interference amongst seasonal epidemics of different respiratory viral infections raises the issue of underlying interference mechanisms, and provides improved prediction of epidemics, easing the planning for control and prevention strategies [10–12]. For example, the timely and accurate observation of the epidemiology of other respiratory viruses may assist with predicting the timing and size of influenza epidemic and adopt opportune preventative measures [13]. Multiple delays in the influenza pandemic in European countries have attracted attention; these delays are presumably linked to epidemics of other respiratory infections [10, 14]. To gain more insight into the interaction between different respiratory viruses and their subtypes, this study investigated the interference amongst seasonal epidemics of six major respiratory illnesses in Hong Kong from 2014 to 2017 based on laboratory surveillance data.

## Methods

### Laboratory surveillance data

The Hong Kong Centre for Health Protection conducts routine outpatient and laboratory surveillance for the detection of pathogens from respiratory specimens [15, 16]. ILI was defined as a fever ≥38 °C and cough or sore throat [17]. Weekly consultation rates of patients with influenza-like illnesses were reported by General Practitioners (GPs, 50 reporting clinics) and Out-patient Clinics (GOPCs, 64 reporting clinics) spread over Hong Kong, amongst which approximately 24% of total outpatient consultations are held by GOPC and 57% by GP [18]. Respiratory specimens in laboratory surveillance were collected from ILI patients in GOPC and GP and predominately from inpatients with acute respiratory diseases [17, 19]. Consequently, a bias for severe cases exists but the seasonality, particularly the peak and trough periods of the virus infection, remain almost unchanged due to this bias. Since February 2014, molecular testing for specimens has replaced viral culture in Hong Kong [20]. Although asymptomatic viral infections can be missed by the surveillance system, the laboratory surveillance reports the weekly numbers of specimens that were confirmed positive for various viruses (only specimens collected prior to antiviral therapy were counted) and is acknowledged to represent the local situation of respiratory virus activities in Hong Kong. We investigated the seasonal trend of viruses from laboratory surveillance reporting in Hong Kong from 2014 to 2017:

i) Influenza virus subtypes A and B;
ii) Parainfluenza virus (PIV) subtypes I, II, III and IV;
iii) Respiratory syncytial virus (RSV);
iv) Adenovirus;
v) Rhinovirus;
vi) Enterovirus.

Nasopharyngeal aspirates or throat swabs were collected to detect enteroviruses. Coronaviruses, a set of respiratory viruses, were not included in our study because they were not routinely tested and data disclosure was not guaranteed in laboratory surveillance in Hong Kong.

### Statistical analysis

From January 2014 to December 2016, the weekly proportions of positive specimens for influenza and other respiratory viruses out of the total number of laboratory-tested specimens were calculated. For seasonality and the occurrence of epidemic comparison, we presented the weekly positive rates of detected viruses together with the total number of detections during the study period for six respiratory viruses. The description and comparison were repeated for four common subtypes of influenza and PIV. To track the annual seasonal trend of the virus, time series was smoothed by calculating the three-week moving average values [12].

In addition, we applied a seasonal linear regression model to estimate the peak timing of respiratory diseases by assuming the existence of annual and semi-annual cycles [21, 22]. The sinusoidal model was used for fitting, because it is widely used in the sequence with distinct periodicity, and each parameter has an explicit explanation [23]. For example, the weekly positive rates of influenza were fitted as follows:

$$ \mathrm{influenza}(t)=a+b\ast \cos \left(2\pi \ast \frac{t}{52.17}\right)+c\ast \sin \left(2\pi \ast \frac{t}{52.17}\right)+d\ast \cos \left(4\pi \ast \frac{t}{52.17}\right)+e\ast \sin \left(4\pi \ast \frac{t}{52.17}\right)+\varepsilon (t), $$

in which influenza(*t*) represents the weekly proportions of influenza-positive cases; *t* is an index for week; a, b, c, d, e are the intercepts and seasonality coefficients; and *ε*(*t*) is assumed to be normally distributed errors. We extracted the annual and semi-annual peak timing by AnnPeakTiming =  − atan(c/b) and SemiAnnPeakTiming =  − atan(e/d) [22, 24]. To obtain confidence intervals on peak timing estimates, we used a block-bootstrap approach which is appropriate to take into account the auto-correlation in time series data [25]. Specifically, we resampled 1000 influenza time series by the bootstrap method and computed the corresponding seasonal coefficients. Confidence intervals were obtained from the 2.5 and 97.5 percentiles of the bootstrap distribution of the seasonal coefficients. The annual peak timing, semi-annual peak timing and confidence intervals on the peak timing estimates of six common respiratory viruses were calculated in this procedure separately.

## Results

**Fig. 1:**
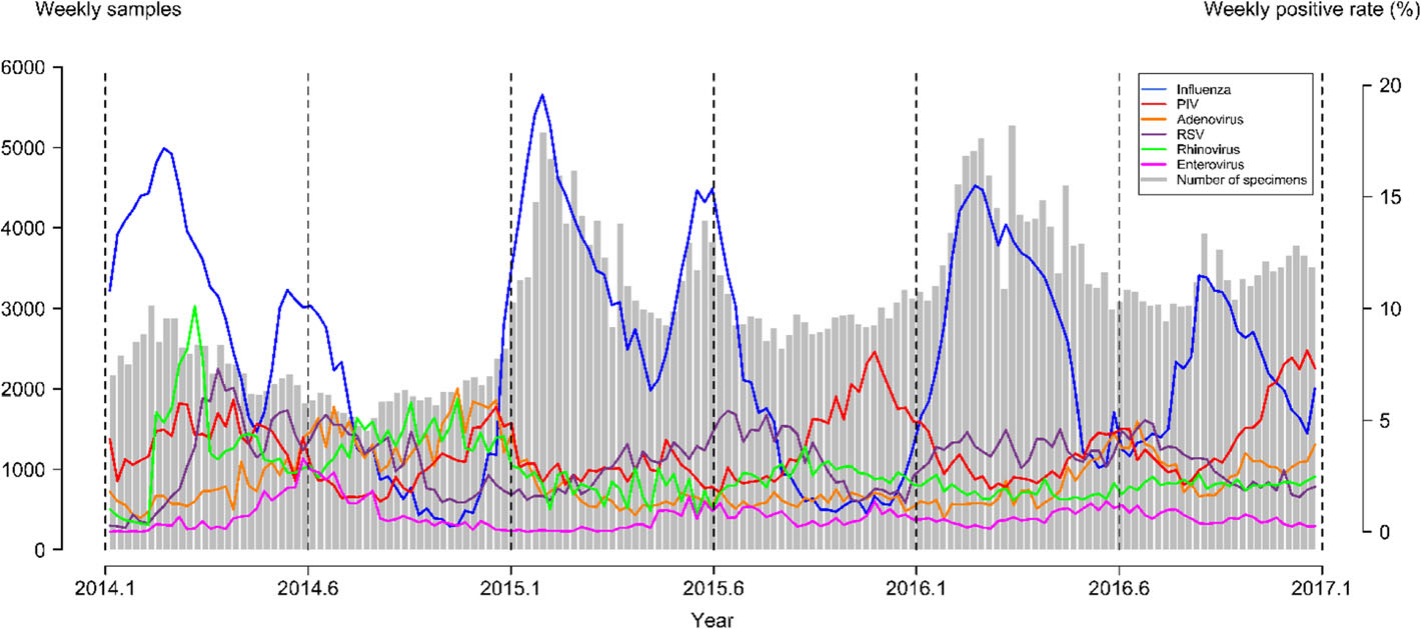
Weekly number of specimens and positivity rates of laboratory-confirmed cases for six viruses in Hong Kong, 2014–2017

This study begins with a summary of the weekly number of respiratory illness infections with viruses routinely tested in the laboratory, including influenza viruses, PIV, RSV, adenoviruses, enteroviruses and rhinoviruses between January 2014 and December 2016 (157 weeks) in Hong Kong. The most laboratory reports were available for influenza A and the least for enterovirus (41,787 and 2875 infections during the entire study period, respectively, Table 1). The time series of weekly laboratory-confirmed positive rates of all considered viruses and the total numbers of the tested specimens are shown in Fig. 1. To facilitate the demonstration of low and high peaks of different viruses in the graph, high peaks of influenza were scaled down (the portion greater than 0.1 was shrunk to 1/3 of the original value, changing the height but not the shape of the epidemic during the period). The weekly total numbers of respiratory samples were cyclical within a year. From January through March, the total numbers of respiratory samples increased to the peak value. A decrease occurred until June, when a small rebound appeared that was lower than the spring peak.

**Table 1:** Summary of weekly laboratory-confirmed samples of common respiratory viruses in Hong Kong, 2014–2017

	Influenza A	Influenza B	Parainfluenza	RSV	Adenovirus	Enterovirus	Rhinovirus
2014–2015							
Total^a^	7756	4255	4031	3308	3463	838	4382
Mean^b^	149	82	78	64	67	16	84
Median^c^	86	60	56	59	65	10	79
Max^d^	637	299	118	140	186	60	258
2015–2016							
Total^a^	20,092	2678	5942	2726	5003	857	3837
Mean^b^	386	52	114	52	96	16	74
Median^c^	158	40	77	45	91	14	76
Max^d^	1962	140	180	134	174	60	119
2016–2017							
Total^a^	13,939	6592	7259	4458	6414	1180	3759
Mean^b^	263	124	137	84	121	22	71
Median^c^	224	38	132	80	119	20	70
Max^d^	905	572	257	159	219	48	103
Total^*^	41,787	13,525	17,232	10,492	14,880	2875	11,978

^*^Total reported cases of laboratory-confirmed respiratory virus samples during 2014–2017

^a^ Annual number of laboratory-confirmed respiratory virus samples

^b,c,d^ Weekly laboratory-confirmed samples of respiratory viruses

**Fig. 2:**
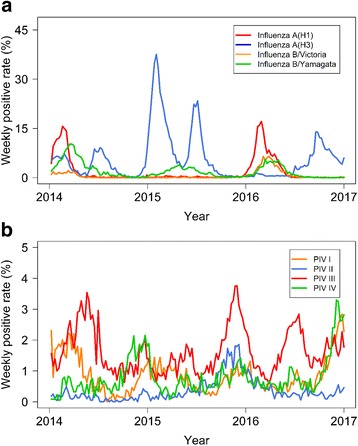
Time series of weekly laboratory-confirmed positive rates of influenza viruses and parainfluenza viruses (PIV) with subtypes in Hong Kong, 2014–2017. **a** influenza viruses; **b** parainfluenza viruses

Winter-to-spring and summer influenza peaks were detected each year from 2014 to 2017, as shown in Fig. 1. By contrast, the peaks of each influenza subtype were not regularly observed, and the peak of the H3 subtype viruses and the H1 subtype viruses of influenza A and subtypes viruses of influenza B were not synchronized (Fig. 2a). During the three-year period, the H3 subtype of influenza A was dominant over the other influenza viruses, particularly in two epidemics of 2015. Meanwhile, the other subtypes of influenza almost disappeared. In the following spring (influenza epidemic of 2016), other influenza virus subtypes became active when the H3 subtype of influenza A was inactive. In addition, the summer influenza epidemic was delayed in 2016 and other types of viruses, except for influenza, were more active compared to their performance during the same period of the previous two years. When the delayed summer peak of influenza arrived, other viruses quickly decreased to activity levels (Fig. 1).

**Fig. 3:**
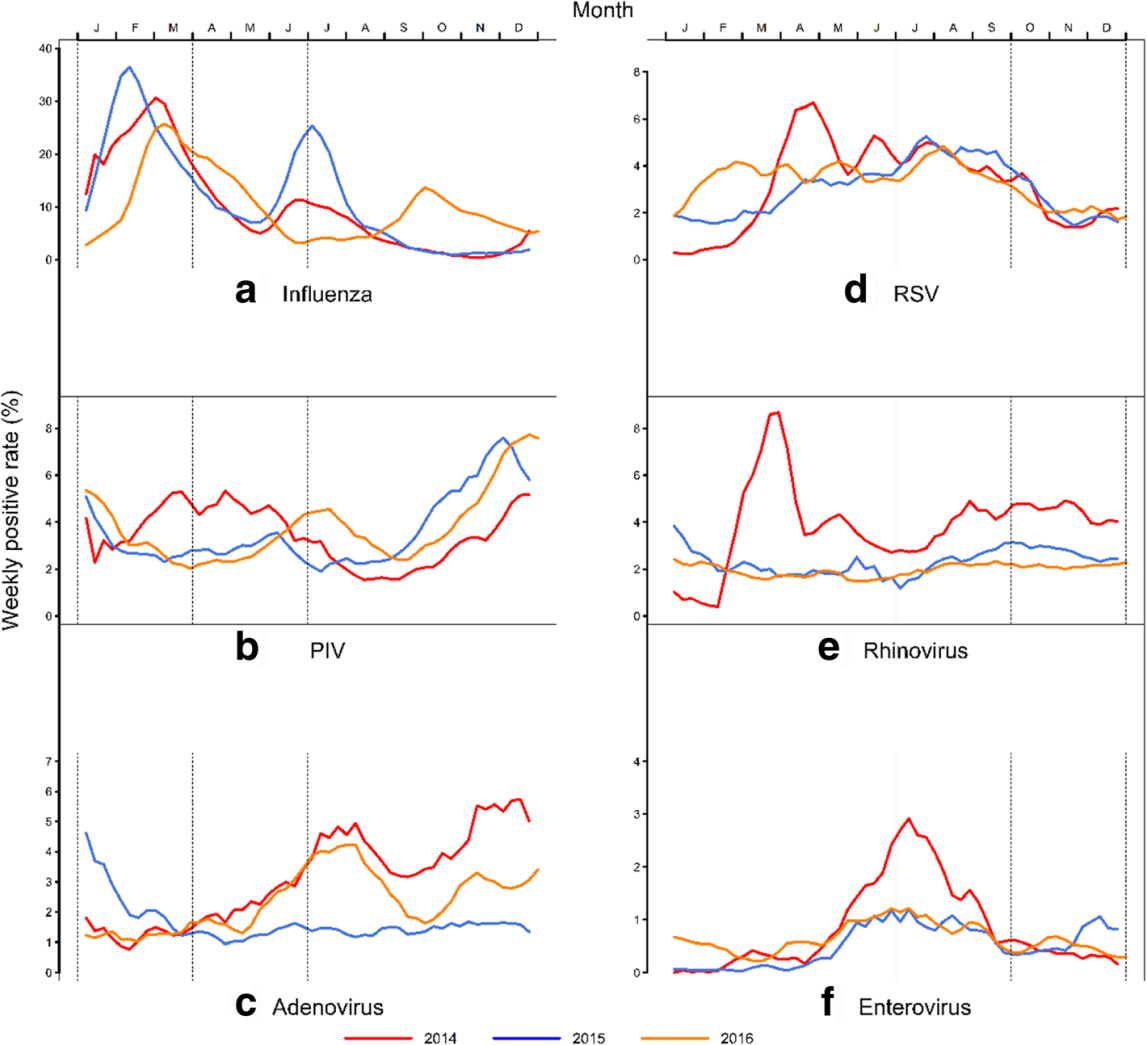
Three-week moving averages of weekly positive rates for six viruses in Hong Kong, 2014–2017. **a** influenza viruses; **b** respiratory syncytial virus; **c** parainfluenza viruses; **d** rhinovirus; **e** adenovirus; **f** enterovirus

The overall weekly detection rates of PIV were lower than those of influenza. However, three annual trends of PIV in Fig. 3 shows that PIV gradually became active from October to December. The average weekly positive rate of the PIV subtype III was higher than the other subtypes during the study period, but it was not always the dominant PIV subtype (Fig. 2b). When influenza viruses were active, the positive rates of PIV were low; the peaks of PIV and influenza viruses were often staggered.

The other three respiratory viruses routinely tested in the laboratory were less active than influenza and PIV (Table 1, Fig. 1). Adenovirus had experienced a high prevalence at the end of 2014, and after a year without obvious fluctuations, the seasonal trend in 2016 was similar to that of 2014 (Fig. 3). The seasonal trends of RSV were similar from year to year, with a low infection rate in the winter that continued to increase until the summer. Rhinovirus had a higher rate of infection in 2014 than in 2015 and 2016, after which the infection rate remained steady. After the rhinovirus epidemic, the following summer influenza epidemics had the lowest impact during the three-year study period (Fig. 1). Although a small number of enteroviruses were detected, the seasonal performance of enterovirus was stable, and an annual summer peak showed up consistently.

**Fig. 4:**
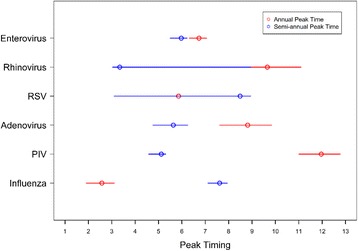
Estimates for annual and semi-annual peak timings of six viruses in Hong Kong, 2014–2017. Open circles represent point estimates from seasonal regression models and horizontal lines represent 95% block-bootstrap confidence intervals based on 1000 replications

The peak timing estimates of influenza virus, PIV, adenovirus and enterovirus are accompanied by their corresponding 95% bootstrap confidence interval (CI) in Fig. 4. The major peak timing estimates of influenza and PIV were focused in February (95%CI: 1.904–3.105) and December (95%CI: 11.004–0.772), whereas the semi-annual peak timing estimates of influenza and PIV occurred in July (95%CI: 7.114–7.94) and late April to May (95%CI: 4.58–5.305). Estimates of the major peak timing of adenovirus and enterovirus were concentrated in the summer months of August (95%CI: 7.623–9.839) and June (95%CI: 5.496–7.057). The semi-annual peak timing estimate of enterovirus was close to the annual peak estimate, which illuminates the unimodal characteristics of enteroviruses. In addition, the CIs of the peak timing estimates of RSV and rhinovirus covered more than half a year, which indicated the implicit peak timings and the stable state of virus activities throughout the year. Notably, most of the viruses peaked in the period of May to early June, which was the inactive season for the influenza virus.

## Discussion

Monitoring a greater variety of viruses with more sample sites will enhance our understanding of viral interaction, interference and geographical transmission. Current multi-regional surveillance data on non-influenza respiratory viruses remain limited, particularly in China and other developing countries. Laboratory surveillance data in Hong Kong from 2014 to 2017 reported that the main pathogenic viruses of acute respiratory infections in this area are influenza viruses. In addition, PIV, RSV, adenovirus and rhinovirus are important components that cause acute respiratory infections. Although enterovirus is often found in the respiratory secretions and stool of an infected person [26, 27], pharynx swab specimens are useful for capturing the seasonality of enterovirus in our study. An enhanced epidemiological surveillance system considering a catch-all approach is more affordable than a single pathogen path, particularly by using residual samples from existing influenza surveillance [3]. To our knowledge, our study is one of the latest studies that focuses on local viral interference in a subtropical city in Asia and contributes new evidence to support the construction of a multi-virus surveillance system.

Seasonal features of the influenza viruses, PIV, enterovirus and adenovirus have been supported by multiyear laboratory-confirmed surveillance data. The influenza viruses usually erupted in February and July, and the summer peaks in July were generally caused by the H3 subtype of influenza A alone. Between the spring and summer peaks of influenza, a rising trend of virus activity contributed by PIV, adenovirus and enterovirus can be observed. After the summer peak of influenza, a PIV epidemic often occurred in November. It is necessary to explore the seasonal epidemics together with mutual interference of influenza and other respiratory viruses. Because seasonality reflects oscillatory changes in infectiousness, which includes contact patterns, pathogen survival, or host susceptibility [28], the correct identification of seasonality can help predict and monitor the epidemic trends of many viruses.

Although the seasonality of influenza viruses is clear, the performances of different subtypes of influenza viruses are not alike. The H3 subtype of influenza A was the main pathogenic subtype of influenza, and the epidemiological trend of influenza subtypes showed that the H3 subtype had an antagonistic effect towards other influenza subtypes to some extent; that is, other influenza subtypes would decrease when the H3 subtype virus was active. In agreement with previous studies, the peaks of influenza A and B coincided less frequently in Hong Kong than in temperate countries [29]. The lineages of influenza B circulated together with influenza A during influenza seasons, with varying incidence from year to year [30].

Previous studies have discovered that viral interferences often occur during virus epidemics and last for a period of time. After the influenza epidemic of 2015, other viruses remained at a fairly low level of infection except for influenza A H3 subtype. When the summer peak of influenza was delayed in 2016, a rise of infections from the other five viruses was observed, indicating an interplay of respiratory virus epidemics. Although the reasons behind this delay were unclear, it can be explained by a potential general anti-influenza virus immunity in the population, triggered by the epidemic in February of 2015. Several immunology studies have confirmed the virus-virus interaction: infections with similar viruses can start producing interferon and other cytokines with similar structures in cells and induce resistance to possible infections by similar viruses [31].

The epidemic of the same virus also has an impact on the subsequent trend of the epidemic. Adenoviruses appeared to be more prevalent than usual in the summer of 2014, followed by an extremely low rate of infection in 2015, with normal levels again in 2016. This occurred presumably because the previous epidemic caused the population to produce a specific antibody to the adenovirus, which led to a significant reduction in the positive rate of adenovirus in the next epidemic season. Some studies on influenza also discovered the phenomenon that after a pandemic of influenza, the subsequent influenza epidemic may be delayed [16].

After the rhinovirus epidemic in 2014, the least obvious summer peak of influenza was noticed from 2014 to 2017 in Hong Kong. The prevalence of rhinovirus may have affected the subsequent flu summer peaks and even inhibited the influenza epidemic. The recent multiple delays of influenza epidemics in European countries had also drawn the attention of researchers to the problem. In Switzerland and some European countries in 2009, swine influenza began to spread after the holidays. Four weeks later, the intensity suddenly declined despite no significant changes of climate and social behavior. The population’s immunity is likely to increase due to infection, but that is unlikely the entire reason, and there were extremely few cases of ILI during the period [10]. Meanwhile, the weekly laboratory-confirmed positive rates of rhinovirus increased significantly. Some retrospective studies have found evidence that rhinovirus infection can reduce adenovirus, PIV and influenza A virus infections [32, 33]. By contrast, although the influenza A epidemic could delay the RSV epidemic and prevent the influenza B virus from occurring at all, it had little effect on the rhinovirus [12]. The mechanism was as follows: once a rhinovirus infection developed, the infected cells began producing interferon and other cytokines, which were similar to those generated by influenza-infected cells [10]. Because the symptoms of rhinovirus infection are often mild or asymptomatic [9], some researchers have suggested that increasing the infection rate of rhinovirus within a certain range may potentially leave the population at a lower risk of influenza epidemics [32].

This study has several limitations. Our results are based on a relatively short time period of three years, which prevents the ability to capture multiyear periodicities of the virus. Our study suggests that virus interference most likely contributed to the changing of virus circulation, but a rigorous statistical analysis assessing the local virus interactions is necessary when evidence is accumulated over a long-term period. Although we cannot determine whether the viruses affect one another biologically or detect the seasonal drivers of risk from the current analysis, the thorough description of the recent surveillance data in Hong Kong still provides solid support on several suspected relationships between influenza and other respiratory viruses by analyzing the regional surveillance data.

## Conclusions

In conclusion, our study captured the seasonal characteristics over the past three years of influenza and five other common viruses that cause respiratory infections in Hong Kong and analyzed the potential interaction amongst the various viruses. As postulated by several short studies, the evidence in Hong Kong demonstrates that viral interference during the epidemics of influenza and other common respiratory viruses might affect the timing and duration of subsequent epidemics of a certain or several viruses. The recent patterns of viral interference are anticipated to be studied in different regions and contributed as advanced guidance to the global prevention and control of respiratory infections.

## Abbreviations

CI: confidence interval
GOPC: general out-patient clinics
GP: general practitioners
ILI: influenza-like illness
PIV: parainfluenza virus
RSV: respiratory syncytial virus
